# Heavy Metal Removal from Aqueous Solutions Using a Customized Bipolar Membrane Electrodialysis Process

**DOI:** 10.3390/molecules29081754

**Published:** 2024-04-12

**Authors:** Samuel Bunani, Gudrun Abbt-Braun, Harald Horn

**Affiliations:** 1 Karlsruhe Institute of Technology, Engler-Bunte-Institut, Water Chemistry and Water Technology, Engler-Bunte-Ring 9, 76131 Karlsruhe, Germany; gudrun.abbt-braun@kit.edu (G.A.-B.); harald.horn@kit.edu (H.H.); 2 CRSNE—Research Center in Natural Sciences and Environment, Faculty of Sciences, University of Burundi, Bujumbura P.O. Box 2700, Burundi; 3 DVGW—Research Center at the Engler-Bunte-Institut, Water Chemistry and Water Technology, Engler-Bunte-Ring 9, 76131 Karlsruhe, Germany

**Keywords:** bipolar membrane, electrodialysis, heavy metals, separation, stack resistance mitigation

## Abstract

Lack of safe water availability and access to clean water cause a higher risk of infectious diseases and other diseases as well. Heavy metals (HMs) are inorganic pollutants that cause severe threats to humans, animals, and the environment. Therefore, an effective HM removal technology is urgently needed. In the present study, a customized bipolar membrane electrodialysis process was used to remove HMs from aqueous solutions. The impacts of the feed ionic strength, applied electrical potential, and the type and concentration of HMs (Cd^2+^, Co^2+^, Cr^3+^, Cu^2+^, and Ni^2+^) on the process performance were investigated. The results showed that feed solution pH changes occurred in four stages: it first decreased linearly before stabilizing in the acidic pH range, followed by an increase and stabilization in the basic range of the pH scale. HM speciation in the basic pH range revealed the presence of anionic HM species. The presence of HMs on anion exchange membranes confirmed the contribution of these membranes for HM removal in the base channels of the process. While no clear trend was seen in the ionic strength solution, the maximum HM removal was observed when 1.5 g/L NaCl was used. The initial HM concentration showed a linear increase in HMs removal of up to 30 mg/L. A similar trend was seen with an increase in the applied electrical potential of up to 15 V. In general, the amount of HMs removed increased in the following order: Cd^2+^ ˃ Ni^2+^ ˃ Co^2+^ ˃ Cu^2+^ ˃ Cr^3+^. Under some operational conditions, however, the removed amount of Cu^2+^, Co^2+^, and Ni^2+^ was similar. The mass balance and SEM-EDX results revealed that the removed HMs were sorbed onto the membranes. In conclusion, this process efficiently separates HMs from aqueous solutions. It showed the features of diluate pH adjustment, reduction in the overall stack electrical resistance, and contribution of anion exchange membranes in multivalent cation removal. The mechanisms involved in HMs removal were diffusion and migration from the bulk solution, followed by their sorption on both cation and anion exchange membranes.

## 1. Introduction

Clean water is one of the precious and important resources for good health for human beings, animals, and even plants. Sustainable management of water resources and access to safe drinking water and sanitation are the principal elements needed to unlock economic growth and productivity and to reduce existing social inequality among people [1,2]. Water resources and water quality in various regions and countries are facing higher pressures because of continuing population growth and industrialization. Many natural water resources are contaminated with heavy metals (HMs) and inorganic anions. This contamination makes water unsafe for drinking. The World Health Organization (WHO) has defined drinking water quality guidelines for HMs and other inorganic contaminants. For instance, antimony, arsenic, barium, boron, cadmium, chromium, copper, fluoride, lead, nitrate, nickel, selenium, and uranium are of higher health concern in drinking water [3]. These contaminants are responsible for various diseases and disabilities in human beings and animals exposed to higher concentrations of water, with a higher risk of environmental pollution [4]. It is a challenging task to remove such dissolved contaminants, and current technologies often need to be operated in multiple stages to achieve effective removal. Current treatment technologies for the removal of HMs and other compounds include adsorption [5], flocculation, ion exchange [6,7,8], membrane filtration such as nanofiltration (NF) and reverse osmosis (RO) [9,10,11] and electromembrane processes such as electrodialysis (ED) [12,13,14], bipolar membrane electrodialysis (BMED) [15,16], and electrodeionization (EDI) [17,18]. The limitations of these technologies are their high operating costs and only partial removal, especially for marginally polluted brackish groundwater/surface water or industrial effluents. The lack of selectivity, low capacity, potential for scaling and fouling, and expensive regeneration requirements are other limitations of most of these decontamination technologies. Electromembrane processes such as electrodialysis (ED), membrane capacitive deionization (MCDI), bipolar membrane electrodialysis (BMED), and electrodeionization (EDI) are reported to be more economically efficient. Moreover, they are well known for their capability of removing and/or recovering ionic species from various water resources. Both techniques contain ion exchange membranes in their modules, which allow a selective passage of ions toward their respective cathode and anode sides under an applied electrical potential as the driving force. Although the main applications of electromembrane-based processes are primary desalination, water purification, and demineralization of food products [19], they are also applied to the processing of solutions containing organic compounds [20], concentrating solutions [21,22], acid/base production, and recovering precious elements from liquid solutions [23,24,25]. In recent years, ED has extended its application in wastewater treatment, chemical processes, and food and drug industries due to the development of new membranes. Moreover, ED is considered a clean production process and is used for the purpose of metal recovery from plating industry effluents [4,26]. In the course of conventional ED application, the process performance is affected by ion exchange material properties, feed solution characteristics, applied electrical potential/current, and the design or geometry of the electrodialyzer. Although ED is a promising technology for the removal of metal ions, the cell resistance increases as a consequence of ion depletion in the diluate compartment. This phenomenon is undesirable in ED. An increase in cell resistance results in higher energy consumption and lower removal efficiency [27,28]. As a consequence of ion depletion near the ion exchange membrane surface, a boundary layer is formed due to the concentration polarization. The concentration polarization phenomenon is somewhat inevitable and directly impacts the ED process performance [29]. Since the ion transport continues from desalting cells to concentrated ones, ion concentrations may reach zero on the membrane surface. The current density at this point of zero-ion concentration is called the limiting current density. The process then evolves in an over-limiting current density situation, where water dissociation occurs to produce H^+^ and OH^–^ ions as new charge carriers. At an over-limiting current density, the ion transport in the membrane boundary layer is assumed to be due to gravitational convection [30], joule heat [31,32], and electric field [33,34]. Many studies have reported strategies for reducing cell resistance. The ion exchange membrane modification approach is the most commonly used strategy to overcome the membrane’s higher resistance and to improve its selectivity.

In the present work, the aim was to set up a new electromembrane process capable of adjusting the pH of the diluate to improve the HM removal efficiency and multivalent selectivity. Based on monovalent and BM features, the ED principle, and the chemistry of HMs in different pH ranges, the designing of a diluate side bipolar membrane electrodialysis (DS-BMED) was undertaken as a new approach to ED configuration modification. The impact of the feed ionic strength (concentration of NaCl), electrical potential, and the type and concentration of HMs on the process performance was investigated. The DS-BMED performance was evaluated based on its capacity to adjust the pH of the diluate, remove HMs and their corresponding anions, remove the electrical conductivity (EC), and maintain a high current flow through the DS-BMED stack.

## 2. Results and Discussion

### 2.1. Impact of Process Parameters on pH Profile in Diluate Compartment

**Figure 1:**
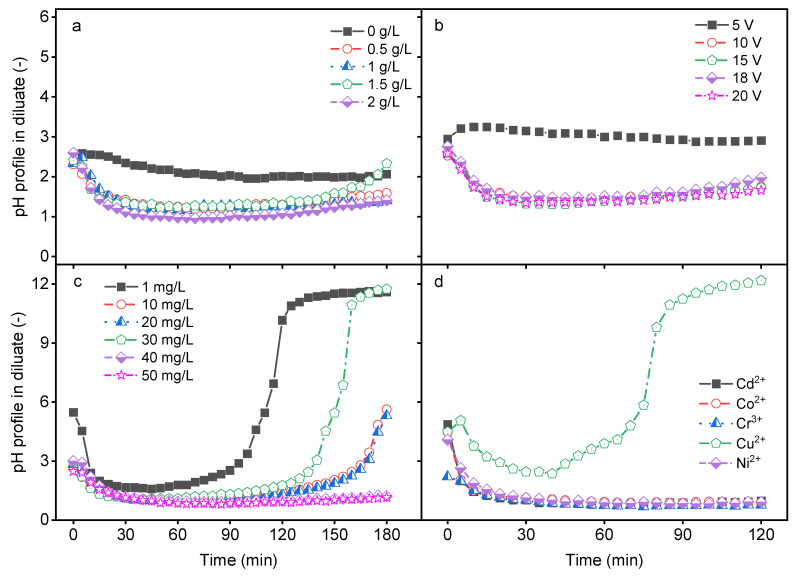
(**a**) Impact of ionic strength (NaCl) on pH in the diluate as a function of time. (**b**) Impact of electrical potential on pH in the diluate as a function of time. (**c**) Impact of the concentration of each HM on pH in the diluate as a function of time. (**d**) Impact of the type of HM on pH in the diluate as a function of time.

In a bipolar membrane (BM), water dissociation occurs at the interface between the cation and anion exchange layers to produce H^+^ and OH^−^ ions. The production rate and the transport of these ions are affected by various factors. The impact of the feed ionic strength, applied electrical potential, HM concentration, and the type of HM on diluate pH change was investigated. As shown in Figure 1a, the pH of the feed solution changed with increasing time with an increase in the ionic strength (*I* = 0–0.034 for NaCl concentrations between 0 and 2 g/L). When DS-BMED was applied to a feed solution containing only HMs and their corresponding anions, no significant change in the pH was observed in the diluate solution. The solution without additional ions (NaCl solution) showed higher electrical resistance, so water splitting in the BM was not promoted. However, the presence of additional ions in the feed reduced the diluate solution resistance and promoted water splitting in the BM, which resulted in a pH change. The pH change in the diluate solution showed three different steps within the time for different salt solutions: a linear pH decrease in the first 20 min followed by pH stabilization in the acidic range, and finally a smooth increase in pH up to the initial pH especially for 1.5 g/L NaCl (Figure 1a). A similar trend of pH change was obtained with an applied electrical potential higher than 5 V (Figure 1b). The HM feed concentration affected the pH of the diluate as well. Depending on the initial concentration of HM, four stages were observed: a decrease and stabilization in the acidic pH range, followed by an increase and stabilization in the alkaline pH range (Figure 1c). Different pH change stages were obtained depending on the type of HM in the feed solution (Figure 1d). All four pH change stages were achieved with only Cu^2+^ as the HMs in the feed and with HM concentrations below 20 mg/L (Figure 1c,d). The other HMs revealed only two stages in the acidic pH range: a decrease and a stabilization (Figure 1d). The results prove that all suspected parameters affected the pH of the diluate compartment. Some of these parameters affected the pH solution in a similar way, while others affected it differently. It can be concluded that the DS-BMED process can contribute to pH adjustment without chemical addition for a known feed solution composition under specific operational conditions.

### 2.2. Impact of pH on HM Species Distribution

Water characteristics, such as pH, chemical composition, component concentrations, ionic strength, temperature, and pressure, determine the speciation of all present components. The HM speciation modeling was performed using the Visual MINTEQ 3.1 software. In the model, a solution containing 1.5 g/L of NaCl and 50 mg/L of each HM and their corresponding anions were considered at 21 °C. The HM species obtained in the acidic pH range (pH = 1–3), at neutral pH (pH = 7), and at basic pH range (pH = 11–13) are summarized in Table 1. The empty cell in Table 1 refers to a species distribution of less than 5% at a given pH. Only a species distribution higher than 5% was reported here (this is why the sum of the species percentage is less than 100%). For pH lower than 3, most of the species of HMs were found to be in the cationic form, which is very important for their transport in electromembrane processes. At neutral pH, although HM species were positively charged to be transported toward the cathode side, they became bigger ions, which reduced their mobility during their transport. The pH in the basic range showed that the species were distributed in both molecular (neutral) and anionic forms (Table 1). Neutral forms of HMs did not contribute to the overall ion transport in electromembrane processes. The presence of the anionic species of HMs suggests that the anion exchange membrane contributed to the removal of HMs during the DS-BMED process (Supplementary Materials).

### 2.3. Impact of Initial Salt Concentration

**Figure 2:**
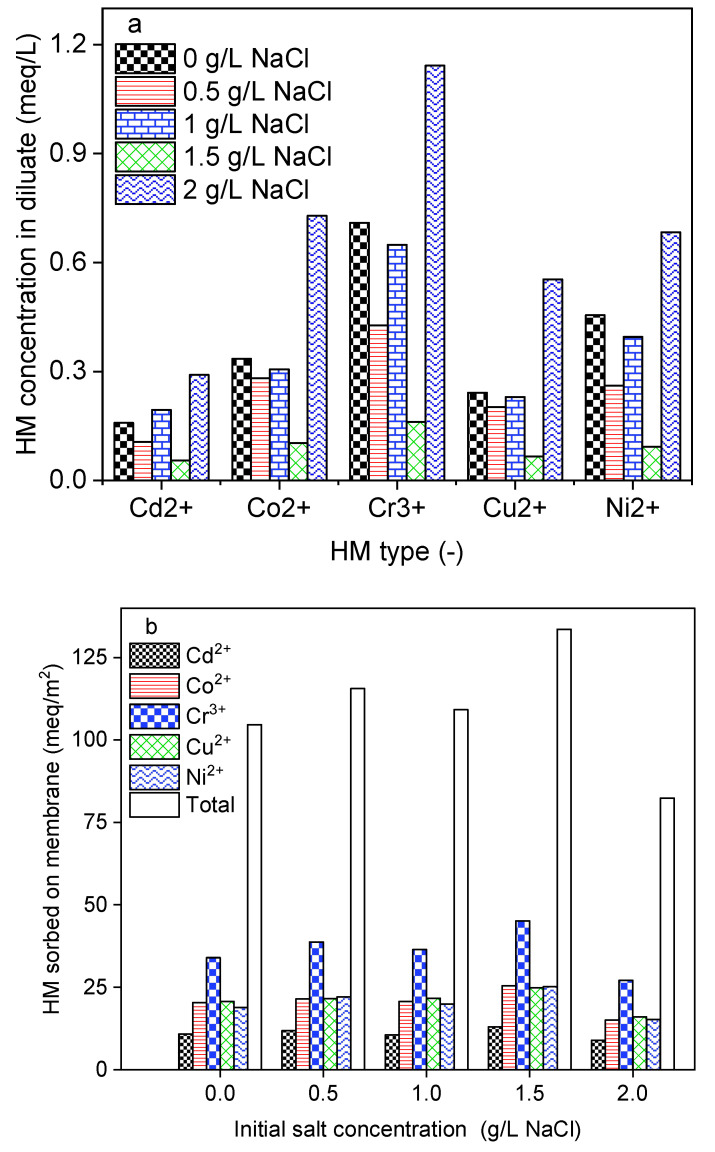
(**a**) HMs in the diluate (feed) at the end of the experiment as a function of initial ionic strength. (**b**) HMs sorbed onto membranes at the end of the experiment as a function of initial ionic strength.

In a natural environment, HMs are found in water resources with different ionic strength backgrounds. To create feed water solutions with various ionic strengths, 50 mg/L of each HM was prepared in 0; 1; 1.5, and 2 g/L NaCl solutions. During this investigation, DS-BMED was set at a constant applied electrical potential of 15 V and the process was run for 180 min. As shown in Figure 2a, HMs in the diluate decreased with salt concentration up to 1.5 g/L of NaCl, which resulted in high removal efficiency. At a salt concentration higher than 1.5 g/L, HMs in the diluate increased sharply. The feed solution containing 1.5 g/L NaCl was found to achieve removal efficiencies higher than 95% for HM. A large portion of the removed HMs was sorbed onto the membranes, as shown in Figure 2b. In contrast to conventional ED, DS-BMED cannot be evaluated based on the EC removal efficiency. The novel process was designed in such a way that in desalting (diluate) channels, the production of H^+^ and OH^−^ from water splitting in BM can maintain a higher EC and consequently reduce the solution resistance. The ionic strength promoted the current flow and prevented water ions from spitting, maintaining it at a higher level. A higher current flow until the end of the process is evidence of the mitigation of the overall stack resistance. In conventional electromembrane-based processes, the current flow declines gradually and this behavior testifies to the increase in stack resistance over time [35]. The H^+^ and OH^−^ contributed in three ways to ensure higher HM removal efficiencies: (i) converting neutral forms of HMs into ionic forms, (ii) reducing the overall stack resistance, and (iii) avoiding multivalent cation precipitation on cation exchange membranes in the process.

### 2.4. Impact of Applied Electrical Potential

**Figure 3:**
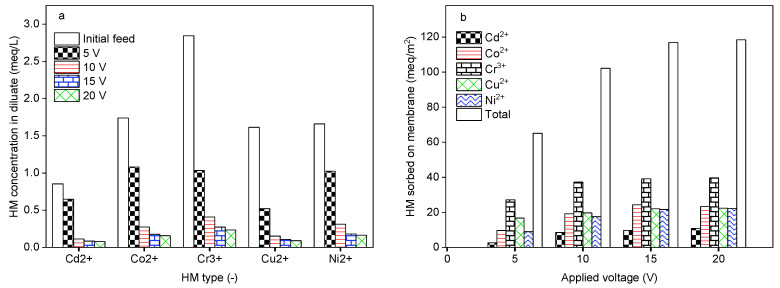
(**a**) HMs in the diluate at the end of the experiment as a function of the applied potential. (**b**) HMs sorbed onto the membrane as a function of the applied electrical potential.

**Figure 4:**
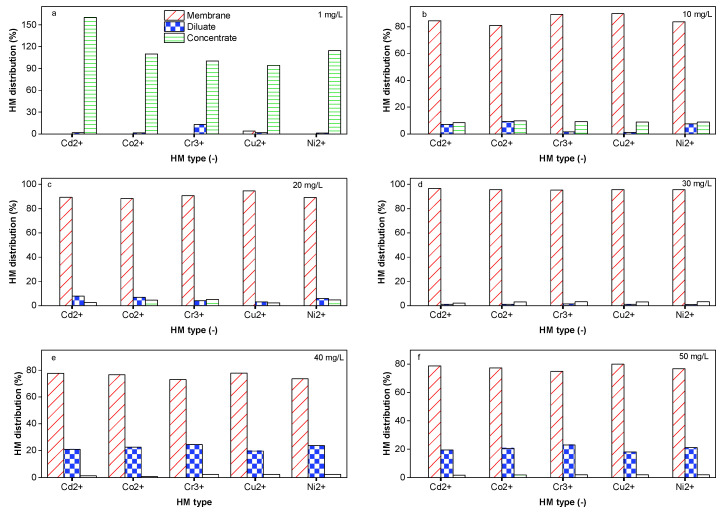
(**a**) HM distribution in (%) at 1 mg/L as the initial concentration of each HM. (**b**) HM distribution in (%) at 10 mg/L as the initial concentration of each HM. (**c**) HM distribution in (%) at 20 mg/L as the initial concentration of each HM. (**d**) HM distribution (in%) at 30 mg/L as the initial concentration of each HM. (**e**) HM distribution in (%) at 40 mg/L as the initial concentration of each HM. (**f**) HM distribution in (%) at 50 mg/L as the initial concentration of each HM.

The influence of the applied electrical potential on the DS-BMED performance was investigated by setting the DS-BMED at 5, 10, 15, and 20 V as the applied voltage for a processing time of 120 min. A feed solution containing 1 g/L NaCl, 50 mg/L Cd^2+^, Co^2+^, Cr^3+^, Cu^2+^, and Ni^2+^ as HMs was used. As shown in Figure 4a, the amount of HMs remaining in the diluate compartment was lower with an applied potential of up to 15 V. Higher removal performances of ≥95% in terms of HMs were achieved at 10 to 15 V. Consequently, the HMs sorbed onto the membrane increased gradually with an applied potential of up to 15 V. The potential higher than 15 V neither improved the product water quality nor the quantity of HMs sorbed onto the membranes (Figure 3a,b). As for other electromembrane-based processes, DS-BMED was affected by the applied driving force. However, DS-BMED has the advantage of reducing the overall stack resistance. Continuous production of H^+^ in acid channels avoided the HM precipitation on the cation exchange membrane, which is an added value of the DS-BMED compared to the current electromembrane processes. Moreover, this feature of DS-BMED helped to convert the HMs anionic form (Table 1), which resulted in HM anionic species being transported toward the anion exchange membrane.

### 2.5. Impact of the Type of HM

Groundwater and/or surface waters and various industrial effluents contaminated with HMs are typically related to human activities, such as industrial processes, electric energy generation, mining, and fuel production. The type of HM in the water depends on the activities being run directly in the surrounding areas. Contaminants can be indirectly transported to water bodies far from other polluted zones. Therefore, it is important to evaluate how each type of HM can be removed by DS-BMED. DS-BMED set at a constant voltage of 15 V was applied to a solution containing 1.5 g/L of NaCl and an initial concentration of 50 mg/L for each HM (Cd^2+^, Co^2+^, Cr^3+^, Cu^2+^, and, Ni^2+^). This process was run for 120 min. According to the data shown in Figure 4a–f, the distributions of HMs between the diluate, membranes, and concentrate at the end of the experiment reveal that Cu^2+^ was highly removed HM, with a removal efficiency of 98%. The lowest removal efficiency was 55% for Cr^3+^. The removal efficiencies of these HMs were in the following order: Cu^2+^ ˃ Cd^2+^ ˃ Co^2+^ ˃ Ni^2+^ ˃ Cr^3+^. Looking at the mechanistic explanation of this rejection order, it seems that neither the ion size, valence, nor their hydration energies can explain it. Both factors act synergistically to contribute to the rejection in different proportions. As can be seen in Table 1, Cu^2+^ was the only HM with a lower percentage of neutral form than the others, especially at basic pH (only 11% of Cu^2+^ was in the form of Cu(OH)_2_ (aq)). This factor should be the main reason for the higher removal performance of Cu^2+^. The other HMs revealed a higher percentage of their neutral forms in the same base region of pH, leading to lower/no mobility of the HM in the basic channels.

### 2.6. Impact of the HM Feed Concentration

**Figure 5:**
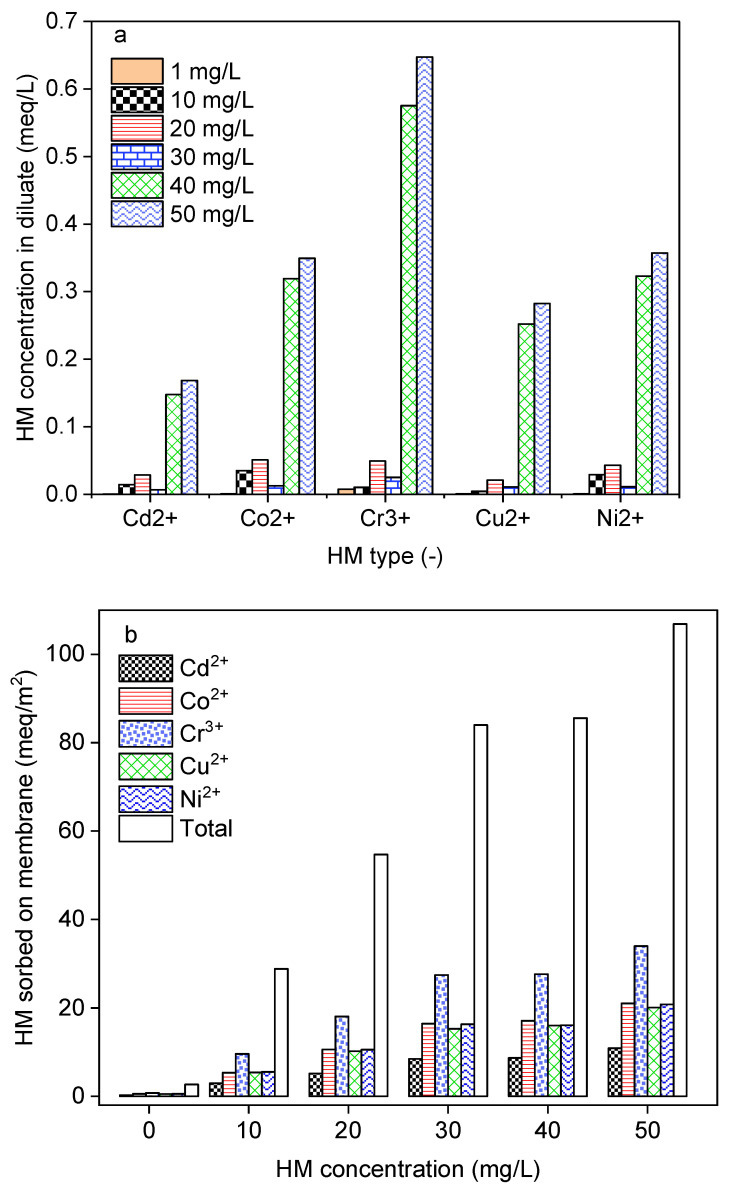
(**a**) HMs in the diluate at the end of the experiment as a function of the initial concentration of each HM. (**b**) HMs sorbed onto membranes as a function of the initial concentration of each HM.

In water treatment processes, the performances in terms of contaminant removal are affected by the chemical composition and concentration. DS-BMED with an electrical potential of 15 V was applied to solutions of 1.5 g/L of NaCl containing HM concentrations in the range of 1–50 mg/L for 180 min. The results shown in Figure 5a revealed that HM concentrations higher than 30 mg/L negatively affect the process in terms of HM removal efficiency. The results obtained from this work showed higher HM removal efficiencies (above 98%) for ED than those reported in the literature [8,12,14,35]. According to the mass balance evaluation, only 1–10% of the removed HMs was transferred to the concentrate compartment. This finding suggests that around 88–97% of the removed portion was sorbed onto the ion exchange membrane (Figure 5b). For this reason, DS-BMED cannot be used to concentrate the HM recovery.

## 3. Methods and Materials

### 3.1. DS-BMED Process Set Up

**Figure 6:**
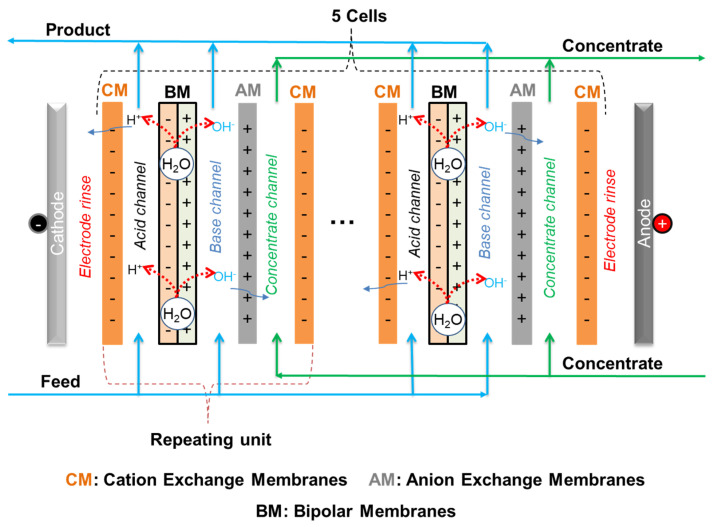
DS-BMED stack flow diagram.

A customized electrodialysis stack with a bipolar membrane, the so-called diluate side bipolar membrane electrodialysis (DS-BMED) (Figure 6), was used for the removal of heavy metals from aqueous solutions. The DS-BMED experiments were carried out using a bench-scale PCCell ED 640 04 model ED cell unit with 3 chamber systems (from PCCell GmbH, Heusweiler, Germany). The DS-BMED stack was completely assembled with 5 cells composed of 3 channels each (acid, base, and concentrate channels) (Figure 6). The standard cation exchange membranes PC SK, bipolar membranes, anion exchange membrane PC Acid 60 (PCA-Polymerchemie Altmeier GmbH, Heusweiler, Germany), and electrodes (anode: Pt/Ir-MMO-coated Ti-stretched metal; cathode: stainless steel) were packed together to build the whole stack. The technical specifications of the ion exchange membranes are given in Table 2. The thickness of the feed and concentrate circuits were ca 1.5 mm and 0.5 mm; respectively. The sizes of the cells used were 110 × 110 mm, and the active area of each membrane was 64 cm^2^. The configuration of the membranes in the DS-BMED stack was CM-BM-AM-CM for a single-cell unit (CM, cationic exchange membrane; AM, anionic exchange membrane; and BM, bipolar membrane). The end membranes on both the cathode and anode sides after assembling 5 cell units were special end cation exchange membranes. The bipolar membranes were integrated into the feed chambers to create acidic and basic channels.

The system configuration allows different feed solution circuits. In the present work, the feed solution inlet was considered as the acidic channel inlet and the acid outlet as the basic channel inlet, whereas the basic outlet was the feed solution outlet. That is, the feed solution circulated first in all acidic channels before the basic channels during the process. The feed solutions containing Cd^2+^, Co^2+^, Cr^3+^, Cu^2+^, Na^+^, and Ni^2+^ as cations and Cl^−^, NO_3_^−^, and SO_4_^2−^ as anions were used. The HM concentrations were prepared at different concentrations (1 mg/L, 10 mg/L, 20 mg/L, 30 mg/L, 40 mg/L, and 50 mg/L) in ultrapure water containing or not some amount of NaCl. The salts used to prepare the HM content were Cd(NO_3_)_2_·4H_2_O, CoSO_4_·7H_2_O, Cr(NO_3_)_3_·9H_2_O, CuSO_4_·5H_2_O, and NiSO_4_·6H_2_O. Except for CoSO_4_·7H_2_O and Na_2_SO_4_, which were purchased from Carl ROTH GmbH, Karlsruhe, Germany and Bernd Kraft GmbH, Oberhausen, Germany respectively, all other chemicals were purchased from Merck Chemicals GmbH, Darmstadt, Germany. The volume of the feed and concentrate solution was 1 L. The concentrate solutions were similar to those of the feed without HM. The electrode solution was prepared by dissolving 50 g/L of Na_2_SO_4_ in ultrapure water. The process was run at constant water flow rates of 20 L/h, 10 L/h, and 120 L/h for the feed, concentrate, and electrode compartments, respectively. The process setup parameters and operational conditions are summarized in Table 3. The temperature was kept constant at around 21 ± 1 °C. The EC, pH, and temperature values were automatically recorded using an ED PC Frontend system. An ICP-OES (inductively coupled plasma–optical emission spectrophotometry) instrument was used for HMs and sodium analyses. The HM content in the membranes was identified using the SEM-EDX method. For HM speciation modeling, Visual MINTEQ 3.1 software was used.

### 3.2. DS-BMED Evaluation Parameters

The performance of the DS-BMED process was evaluated based on pH profiles in the diluate compartment and removal efficiencies of HM. The removal efficiencies, *R* (%), for HMs were evaluated following Equation (1). The mechanisms involved in the process were discussed based on the mass balances deducted from the mass of HMs in the diluate, concentrate, and membrane at the beginning and end of the experiments (Equation (2)). The ionic strength of the solutions, *I*, was calculated using Equation (3) based on the concentration of NaCl.

(1) $$R=\left(1-\frac{C_{Dt}}{C_{D0}}\right)\times 100$$

(2) $$m_{HM}=m_{D0}-m_{Dt}-m_{Ct}$$

(3) $$I=\frac{1}{2}\sum_{i=1}^{n}C_{i}Z_{i}^{2}$$

where $C_{D0}$ (mg/L) and $C_{Dt}$ (mg/L) are the initial and final concentrations in the sample in the diluate compartment; $m_{M}$, $m_{D0}$, $m_{Dt},$ and $m_{Ct}$ are the masses of HMs (mg) fixed in the membrane in the diluate compartment at the initial and final times of the process, and in the concentrate compartment at the end of the process, respectively. In Equation (3), *n* is the number of ions present in the solution, $C_{i}$ and $Z_{i}$ *i*th ion, respectively.

## 4. Conclusions

In the present work, a novel diluate side bipolar membrane electrodialysis (DS-BMED) was used for the removal of HMs from aqueous solutions. The ionic strength of the feed solution, applied driving force, and the type and concentration of HMs were found to affect the process efficiency in terms of HM removal. The findings from this study revealed the ability of the process to convert HMs into an anionic form, which leads to the contribution of anion exchange membranes in HM removal. DS-BMED achieved higher HM removal performances than other electromembrane-based processes already reported in the literature. In contrast to the current process, it cannot be used to recover HM. In terms of the removal mechanisms involved in the process, HM ionic species diffusion and migration from the bulk solution and their sorption onto the membrane are the principal mechanisms. This process is a good strategy for adjusting the pH of the diluate, converting HMs into anionic forms, mitigating the overall stack resistance, and avoiding the precipitation of HMs on cation exchange membranes. However, it cannot be evaluated based on the electrical conductivity (EC) removal since it continuously maintains the EC of the diluate at a high level.

## Figures and Tables

**Table 1:** HM species distribution in % as a function of pH (solution containing 1.5 g/L NaCl and 50 mg/L of each HM with their corresponding anions at 21 °C).

Type of HM	pH	1	2	3	7	11	12	13
	HM Species							
Cd^2+^	Cd^2+^	51.3	44.2	42.9	41.9			
	CdCl^+^	45.0	49.2	49.3	49.5			
	CdOH^+^					11.1		
	Cd(OH)_2_ (aq)					80.3	88.1	39.7
	Cd(OH)_3_^−^						10.4	50.0
	Cd(OH)_4_^2−^							10.2
Co^2+^	Co^2+^	98.3	94.1	92.0	90.5			
	CoSO_4_ (aq)		5.2	7.2	8.6			
	Co(OH)_2_ (aq)					96.1	80.5	28.0
	Co(OH)_3_^−^						19.4	72.0
Cr^3+^	Cr^3+^	97.0	84.4	74.2				
	CrSO_4_^+^		14.7	19.1				
	Cr_2_(OH)_2_^4+^				7.30			
	Cr_3_(OH)_4_^5+^				69.0			
	Cr(OH)_2_^+^				18.5			
	Cr(OH)_3_ (aq)					70.8	19.4	2.20
	Cr(OH)_4_^−^					29.2	80.6	97.8
Cu^2+^	Cu^2+^	96.4	91.6	89.4	38.7			
	CuSO_4_ (aq)		5.7	7.9				
	Cu_2_(OH)_2_^2+^				25.3			
	Cu_3_(OH)_4_^2+^				24.6			
	CuOH^+^				6.0			
	Cu(OH)_3_^−^					87.1	94.0	62.2
	Cu(OH)_4_^2−^							37.7
	Cu(OH)_2_ (aq)					11.3		
Ni^2+^	Ni^2+^	98.2	94.0	91.9	90.5			
	NiSO_4_ (aq)		5.2	7.2	8.6			
	Ni(OH)_2_ (aq)					45.3	7.6	
	Ni(OH)_3_^−^					54.1	92.3	99.2

**Table 2:** Technical specifications of used ion exchange membranes [24].

Membrane	PC SK	PC Acid 60
Ion exchange form	Cation exchange membrane (CM), sodium	Anion exchange membrane (AM), chloride
Membrane type	Strongly acidic (sulfonic acid)	Strongly alkaline (ammonium)
Transference number:	>0.95	
KCl (0.1/0.5 M) ^a^		>0.95
Acid (0.7/3 M) ^b^		0.55
Thickness (mm)	0.16–0.20	0.16–0.20
Water content (wt%)	~9	~17
Ion exchange capacity:	n/a	
Strong basic (meq/g)		ca 1.14
Weak basic (meq/g)		ca 0.45
Electrical resistance (ohm cm^2^)	~2.5	~2
Burst strength (kg/cm^2^)	4–5	4–5
Operating maximum temperature (°C)	50	60
PCcell bipolar type: PC bip		
Water splitting voltage (V)	1.2–2.2	
Water splitting efficiency (%)	>95	
Operating maximum temperature (°C)	40	
Thickness (mm) ^a^	0.2–0.35	

^a^ Calculated from potentiometric measurements; ^b^ observed current efficiencies.

**Table 3:** Investigated parameters and operational conditions.

	Operational Conditions						
	Sample						
Parameters	NaCl (g/L)	Voltage (V)	Type of HM	Concentration of HM (mg/L)	Volume (L)	Feed Flow Rate (L/h)	Electrode Solution
Ionic strength	0	15	Cd^2+^, Co^2+^, Cr^3+^, Cu^2+^ and Ni^2+^	50	1	20	Na_2_SO_4_ (50 g/L)
	0.5						
	1						
	1.5						
	2						
Electrical potential	1	5					
		10					
		15					
		20					
Type of HM	1.5	15	Cd^2+^				
			Co^2+^				
			Cr^3+^				
			Cu^2+^				
			Ni^2+^				
Concentration of HM			Cd^2+^, Co^2+^, Cr^3+^, Cu^2+^ and Ni^2+^	1			
				10			
				20			
				30			
				40			
				50			

## Data Availability

Data are unavailable due to privacy or ethical restrictions.

## Supplementary Materials

The following supporting information can be downloaded at: https://www.mdpi.com/article/10.3390/molecules29081754/s1, Figure S1: SEM-EDX images of virgin and used membranes.
