# Supplementary material for: Heavy Metal Removal from Aqueous Solutions Using a Customized Bipolar Membrane Electrodialysis Process
Source: Molecules. 2024 Apr 12;29(8):1754. doi: 10.3390/molecules29081754 (PMC11052098; doi:10.3390/molecules29081754)
Supplement: Supplementary file 1 [file molecules-29-01754-s001.zip › molecules-2866554-supplementary.pdf]

Supplementary materials

Figure S1: SEM-EDX images of virgin and used membranes

Virgin anion exchange membrane (AEM)

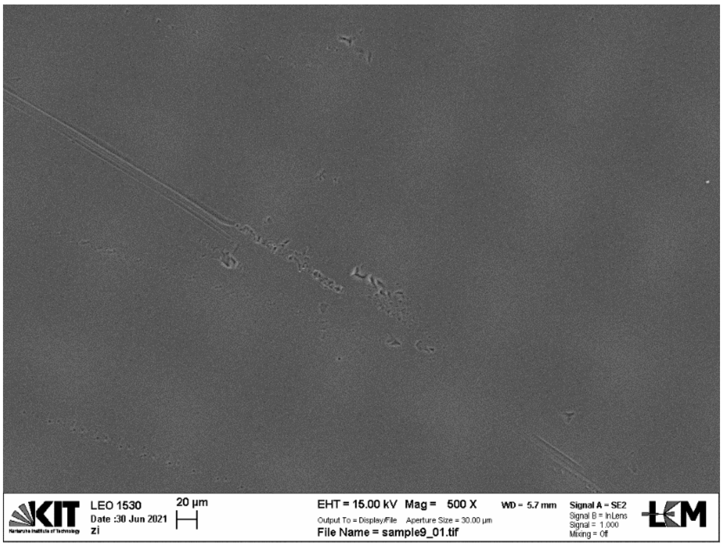

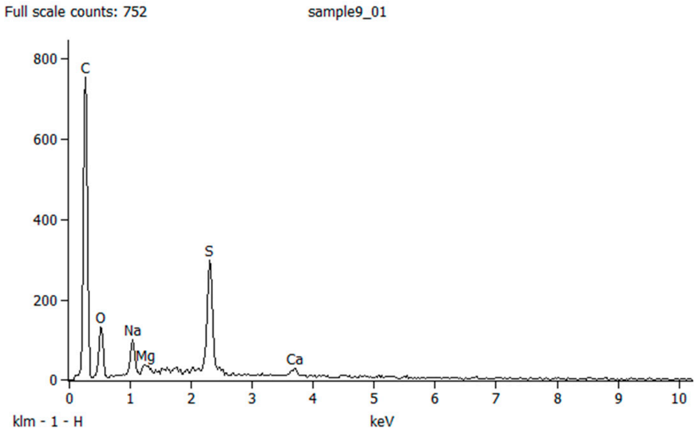

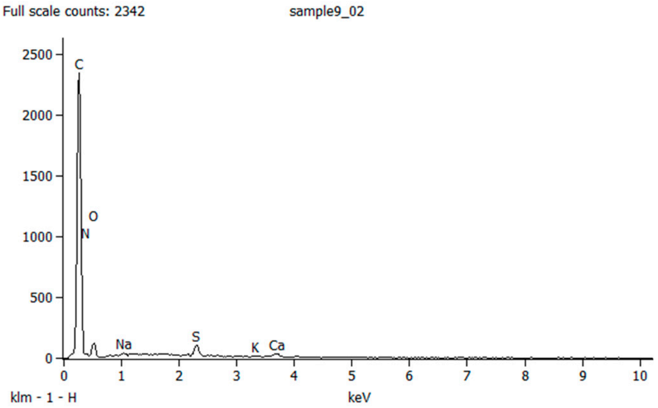

Virgin cation exchange membrane (CEM)

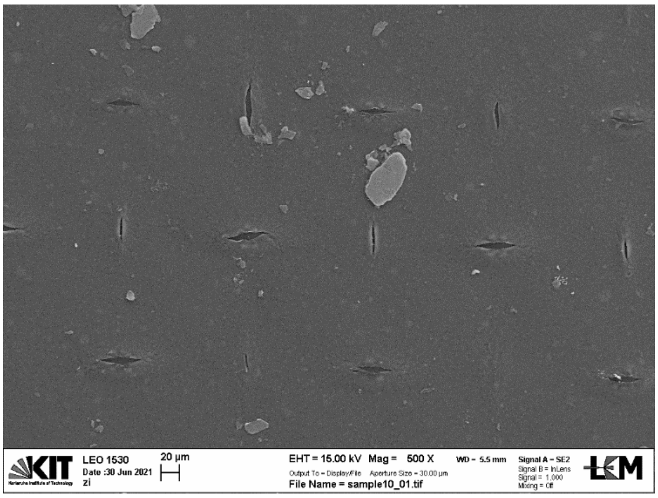

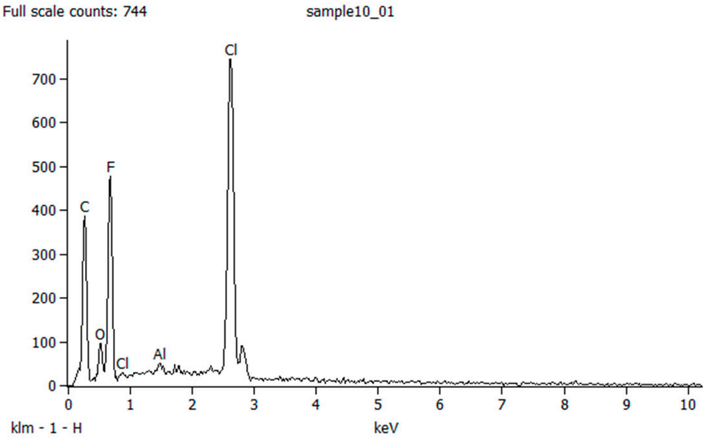

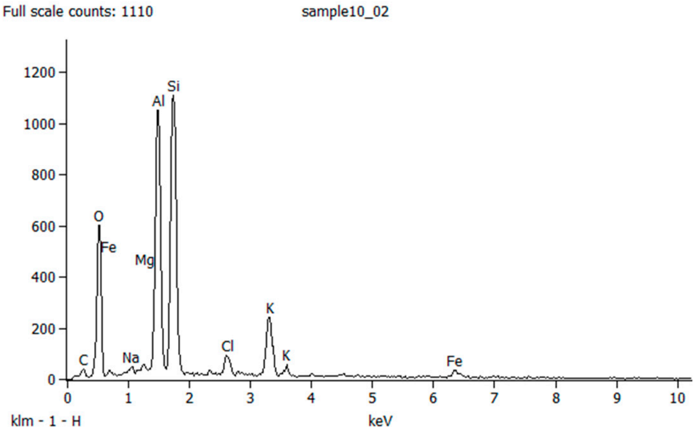

Used Membranes

1. Used AEM+ heavy metal (HM)

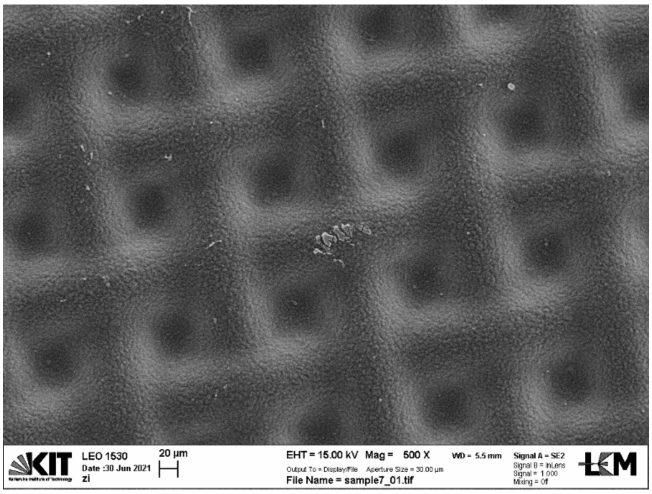

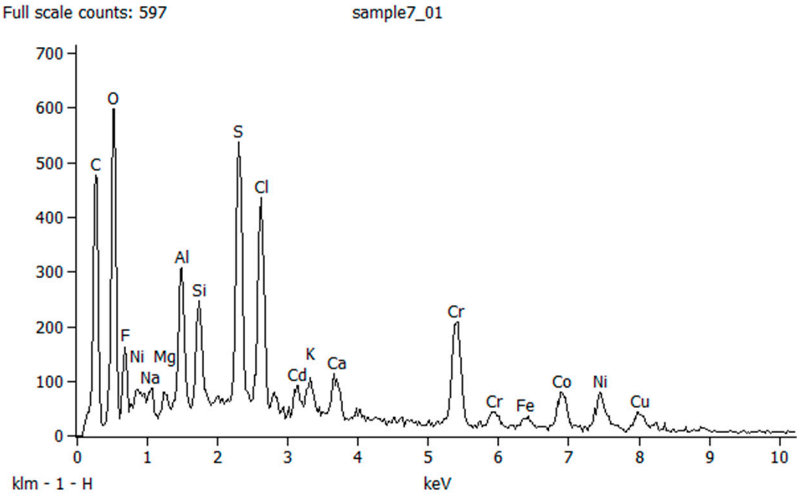

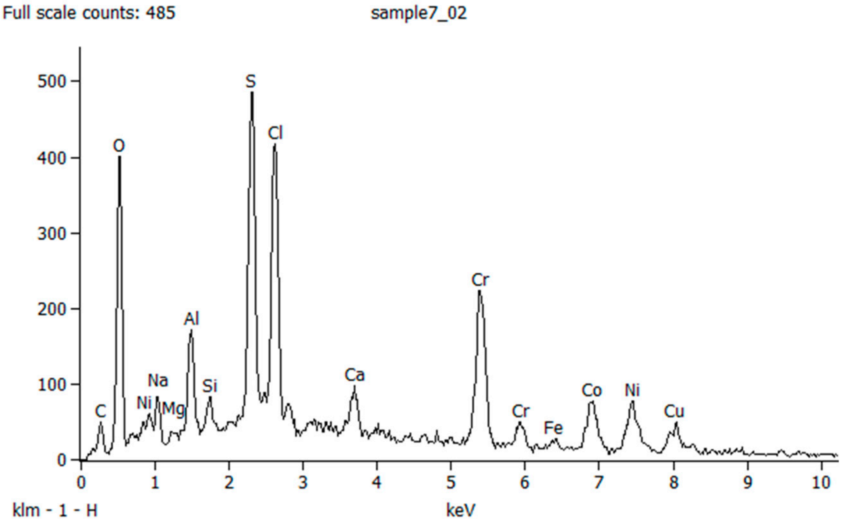

2. Used CEM + heavy metal (HM)

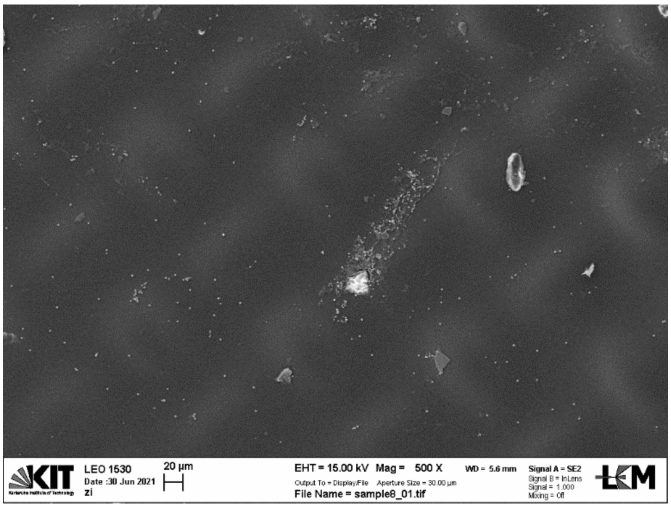

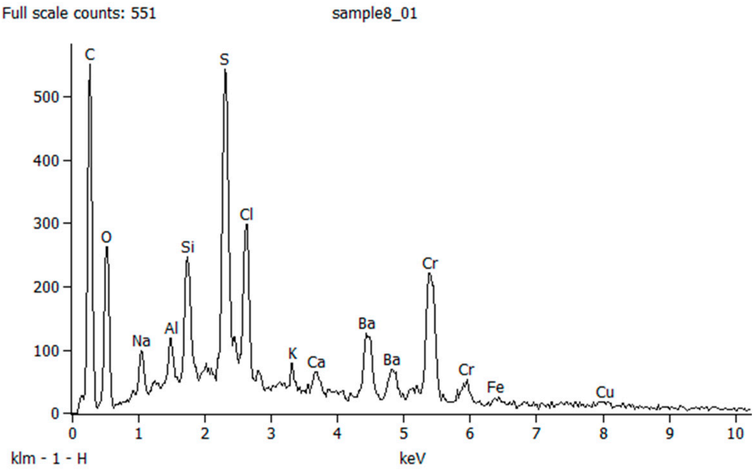

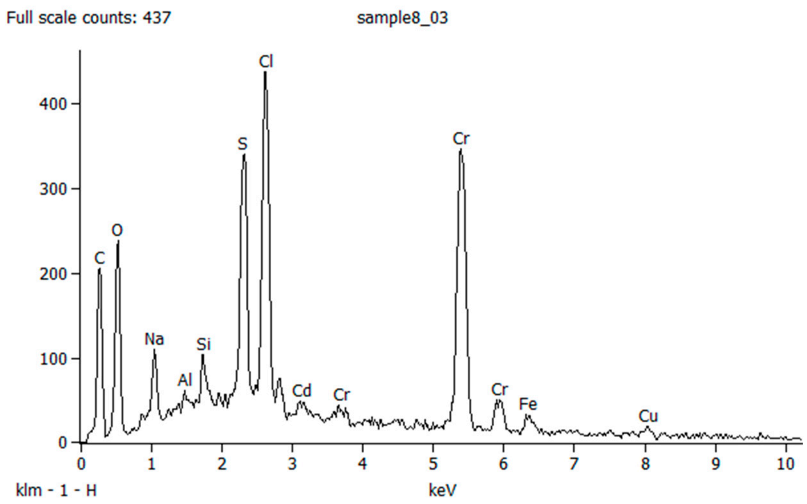

Full scale counts: 437
sample8_03
C
O
Na
Al
Si
S
Cl
Cd
Cr
Cr
Cr
Fe
Cu
0
100
200
300
400
0
1
2
3
4
5
6
7
8
9
10
klm - 1 - H
keV
